# The photosensitizer-based therapies enhance the repairing of skin wounds

**DOI:** 10.3389/fmed.2022.915548

**Published:** 2022-08-11

**Authors:** Xiaoying Ning, Gang He, Weihui Zeng, Yumin Xia

**Affiliations:** ^1^Department of Dermatology, The Second Affiliated Hospital of Xi’an Jiaotong University, Xi’an, China; ^2^State Key Laboratory for Strength and Vibration of Mechanical Structures, Frontier Institute of Science and Technology, Xi’an Jiaotong University, Xi’an, China; ^3^Xi’an Key Laboratory of Sustainable Energy Materials Chemistry, Frontier Institute of Science and Technology, Xi’an Jiaotong University, Xi’an, China

**Keywords:** photosensitizer-based therapy, wound repair, skin, anti-infection, immunomodulatory

## Abstract

Wound repair remains a clinical challenge and bacterial infection is a common complication that may significantly delay healing. Therefore, proper and effective wound management is essential. The photosensitizer-based therapies mainly stimulate the photosensitizer to generate reactive oxygen species through appropriate excitation source irradiation, thereby killing pathogenic microorganisms. Moreover, they initiate local immune responses by inducing the recruitment of immune cells as well as the production of proinflammatory cytokines. In addition, these therapies can stimulate the proliferation, migration and differentiation of skin resident cells, and improve the deposition of extracellular matrix; subsequently, they promote the re-epithelialization, angiogenesis, and tissue remodeling. Studies in multiple animal models and human skin wounds have proved that the superior sterilization property and biological effects of photosensitizer-based therapies during different stages of wound repair. In this review, we summarize the recent advances in photosensitizer-based therapies for enhancing tissue regeneration, and suggest more effective therapeutics for patients with skin wounds.

## Introduction

Skin is the most important barrier of human body to resist external invasion. Wound healing after skin injury consists of a serial of intricate processes involving multiple cells, cytokines, inflammatory responses (1). It is a dynamic and continuous process, with overlapping stages, including hemostasis, inflammation, proliferation, and remodeling (2, 3). Inflammation is the initial stage of wound healing, and it occurs almost simultaneously with hemostasis. As the inflammatory response overlaps, various cytokines and growth factors activate and induce the proliferation, migration, and differentiation of repair cells, resulting in a series of biological behaviors, including epithelialization, angiogenesis and collagen accumulation (3). With the differentiation of fibroblasts into myofibroblasts, continuous tissue remodeling is carried out to participate in wound contraction and restore the continuity of skin (2). Wound healing can be affected by many factors, among which the most common challenge is bacterial infection. Bacteria infection could prolong the inflammation period and interfere with re-epithelialization and collagen deposition, affecting wound healing (4–7). For a long time, antibiotics have been empirically used to prevent wound infections. Drug-resistant bacteria is increasing globally, and new strategies that can simultaneously control infection and promote wound healing without developing drug-resistant bacteria are urgently needed. Recently, several studies have clearly demonstrated the beneficial effect of the photosensitizer-based therapies in the healing of infected wounds (8–11).

Photodynamic therapy (PDT) is the most common type of the photosensitizer-based therapies. It was accidentally discovered in 1900 by Oscar Raab. He recognized that micro-organisms were killed when exposed to light, but not when they were kept in the dark (12). Later on, this approach was clinically used in cancer therapy and antimicrobial application. In the past few decades, the research has overwhelmingly focused on the treatment of cancer and it has become a safe modality of tumor ablation for multiple cancer indications. To date, the currently approved dermatological indications of PDT include actinic keratosis, Bowen’s disease (squamous cell carcinoma *in situ*), and basal cell carcinoma (13). Anti-tumor regimens require lethal doses of photosensitizer (PS) and light, while low-dose treatment have immunomodulatory, antibacterial, and regenerative properties (14, 15). In recent years, the interest in the anti-infective effects of PDT has been revived. It is proposed as a therapy for bacterial, fungal, and viral infections, in which case it is described as antimicrobial PDT (aPDT), or photodynamic inactivation (16). As a new treatment option for inflammatory and infectious diseases, it has been successfully used in skin diseases, such as acne, viral warts, and leishmaniasis (14, 17). Topical PDT is widely used in skin diseases, and it is also an effective method for skin rejuvenation due to the immunomodulatory and antimicrobial effects (17–22).

## The types, principles, advantages, and disadvantages of the photosensitizer-based therapies

**FIGURE 1:**
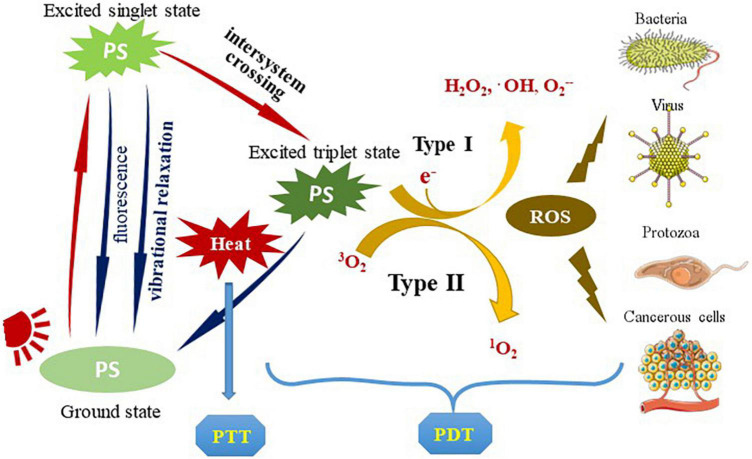
The types and principles of the photosensitizer-based therapies. PS is transformed into an excited singlet state after being irradiated by light. Partially return to the ground state by emitting fluorescence or non-radiative decay, or it can undergo intersystem crossing to the excited triplet state. It partially returns to the ground state by a radiative or non-radiative process, which is usually accompanied by photothermal conversion (used for PTT). The generation of ROS is subsequently promoted by two types of chemical reactions: type I mechanism generates free radicals and radical ions through electron transfer reactions; type II mechanism involves the reaction of PS with molecular oxygen, where the energy is transferred to the triplet ground state molecular oxygen to generate singlet oxygen. ROS acts on pathogenic microorganisms or tumor cells to produce photodynamic effects (used for PDT). PS, photosensitizer; PTT, photothermal therapy; ROS, reactive oxygen species; PDT, photodynamic therapy.

The photosensitizer-based therapies include the following two main approaches: PDT and photothermal therapy (PTT) (Figure 1). In PTT, photothermal agents absorb energy when irradiated with light of a specific wavelength and transform into the excited singlet state. Then they undergo vibrational relaxation and return to the ground state, during which process enhance the heating in the local area (23). Unlike the local thermal damage caused by PTT, PDT induces cytotoxicity based on the production of reactive oxygen species (ROS). PDT involves three important components: light, PS and molecular oxygen. The PS is preferentially absorbed by rapidly dividing cells (such as microorganisms and tumor cells), and converted into excited singlet state after light irradiation. It partially returns to the ground state by emitting fluorescence or non-radiative form of decay, and can also undergo intersystem crossing to the excited triplet state. The generation of ROS is subsequently promoted by two types of chemical reactions: type I mechanism refers to the PS-induced generation of free radicals and free radical ions through electron transfer reactions; type II mechanism involves the reaction of the PS with molecular oxygen, where the energy is transferred to the triplet ground state molecular oxygen to generate singlet oxygen (11, 15, 23–26).

Properties, such as non-invasiveness, spatiotemporal selectivity, the possibility of the parallel application with other treatment, and good therapeutic effect make PDT and PTT become new options for tumor treatment (23). Compared with common antimicrobials, aPDT offers several advantages, including multi-target process, broad antibacterial spectrum, selective cytotoxicity against bacteria, and high bactericidal efficiency. Generally, irradiation is limited to the infected site with no systemic toxicity (24, 27). Another important advantage is that aPDT is independent toward the pattern of bacterial resistance to antibiotics, showing great potential for resistant bacteria (27, 28).

However, there are some side effects. The penetration of light through biological tissue is limited, meaning that PTT and PDT are generally ineffective for deep lesions. PTT may lead to a heat-shock response (23). PDT is generally well tolerated. Pain is the main adverse effect, which can be alleviated by adjusting the irradiation regimen (29). Other phototoxic lesions also manifest as erythema, edema, and sometimes urticaria, which usually subside quickly within a few days (14, 23, 30). Persistent erythema may develop for several months after treatment (31). Erosions, infection, and sterile pustules are also uncommon adverse effects (32, 33). Long-term adverse effects, such as pigment changes, scarring, are less common (31, 34). However, these drawbacks do not prevent PDT from becoming a promising non-invasive treatment for tumor and infection.

## Anti-infection effects of the photosensitizer-based therapies

Antibiotic resistance is increasing globally. As a non-antibiotic process, the photosensitizer-based therapies are considered as attractive alternative treatment for microorganism eradication. ROS and singlet oxygen generated during the photodynamic process can lead to death of microorganisms by damaging cell membranes, changing the structure of microsomes and impairing certain functions, such as protein cross-linking and oxidative damage to nucleic acids (25, 27, 35, 36). A biofilm is a sessile microbial community that shelters microbes from a variety of environmental assaults, and biofilm-related pathogens can tolerate up to 1000-fold higher levels of antimicrobial agents (26, 37–39). In the subsequent sections, we describe the effective elimination of microorganisms, including bacteria, fungi, protozoa, and viruses, and their biofilms by photosensitizer-based therapies.

On human skin samples *ex vivo*, aPDT with SAPYR [2-((4-pyridinyl)methyl)-1H-phenalen-1-one chloride] (1 mM, 20 mW/cm^2^ for 25 min, light dose of 30 J/cm^2^) reduced the viability of methicillin-resistant *Staphylococcus aureus* (MRSA) by 3.9 ± 0.3 log_10_ orders. aPDT can effectively and safely kill bacteria on the skin surface, and it may have the potential to decolonize the skin *in vivo* (40). The photokilling potential of tribenzoporphyrazines had been assessed *in vitro* at 10 μM concentration and light dose of 30 J/cm^2^, aPDT reduce the growth of *Streptococcus pyogenes* by over 4.7 log_10_ and *Staphylococcus epidermidis* by over 5.7 log_10_, *S. aureus* growth by over 5.9 log_10_, as well as MRSA growth by 5.1 log_10_ (41). PTT has also been found to assist in the efficient killing of multidrug-resistant bacteria (39, 42). Kun Zhou reported a PS for synergistic aPDT and PTT, and it showed high efficiency in killing MRSA in a concentration-, irradiance-, and irradiative time dependence manner *in vitro* (25). Six *S. aureus* strains were treated with aPDT [5 μM photosensitizer 5,10,15,20-tetrakis(1-methylpyridinium-4-yl) porphyrin tetra-iodide and 40 W/m^2^ irradiance] and highly virulent strains were found to be more susceptible to aPDT than low virulent strains. Two virulence factors, free coagulase and enterotoxins A and C, were affected by aPDT, and the amount of active enterotoxin A and C was reduced at least 68%, indicating that aPDT is not only effective in inactivation of microorganism, but also in the degradation of the external virulence factors after release. Further, resistance did not developed after 10 consecutive cycles of treatment (43).

*Trichophyton rubrum* is the most common dermatophyte, and aPDT mediated by methylene blue (MB) could inhibit both spores and hyphae of *T. rubrum in vitro*, and the spores were more sensitive. The antifungal efficacy was positively correlated with the concentration of MB and light dose (44). Photodynamic effects on the viabilities of the biofilm of dermatophytes with employment of 0.1 mM MB and light-emitting diode (LED) (100 mW/cm^2^ for 10 min, total light dose of 60 J/cm^2^) were evaluated *in vitro*, and the colony forming units were reduced up to 4.6 log_10_ against the biofilms formed by *T. rubrum*, 4.3 log_10_ and 4.7 log_10_ against the biofilms formed by *Trichophyton mentagrophytes* and *Microsporum gypseum*. Additionally, the biofilm of dermatophytes became more susceptible to antifungal drugs after aPDT (37). The cell wall of *Candida albicans* is the main binding site of porphyrin-based PS. The cell membrane becomes permeable after irradiation, and PS can penetrate through these defects and crack into the cytoplasm, leading to cell death. Since the mechanism of action appears to be located in the cell wall and membrane, rather than in the cytoplasm, it is unlikely that *C. albicans* can provide resistance to aPDT (45). Overall, aPDT is an attractive alternative for fungal infections and is not prone to drug resistance.

Human papillomavirus infection is linked to cervical cancer and genital condyloma acuminatum. It has been reported that 5-aminolaevulinic acid-based PDT (ALA-PDT) inhibited the proliferation of HeLa cells and effectively reduced the viral load in a time-dependent and dose-dependent manner (46). *Acanthamoeba* is a protist pathogen that can cause blinding keratitis and fatal granulomatous amebic encephalitis. Mannose-conjugated porphyrins selectively targeted the surface membranes of pathogenic *Acanthamoeba* organisms, resulting in amebicidal effects and blocked excystation *in vitro* (47). 85 μM curcumin with light dose of 8 J/cm^2^ (450 nm, 36 mW/cm^2^ for 222 s) reduced the viability of *Leishmania* promastigotes, and it caused obvious changes in the cell morphology and induced apoptosis (48). Irrespective of bacteria, fungi, virus or protozoa, aPDT shows excellent anti-infective properties.

The clinical application of PDT as an anti-infective agent is still at a relatively early stage, and it seems to be effective in the treatment of superficial and local infections, including superficial and subcutaneous skin infections, dental diseases, ventilator-associated pneumonia, and gastric infections (49). Wound infection is the most common application. A total of 32 patients who had chronic ulcers were enrolled and randomized into placebo group or aPDT group mediated by PPA904 (3,7-bis (N, N-dibutylamino) phenothiazin-5-ium bromide). As a result, a reduction in bacterial load immediately post-treatment was observed in aPDT group (50). Another compelling application is acne vulgaris. It has been reported that PDT with 8% methyl aminolaevulinate (MAL), illuminated by 635 nm red light at light dose of 37 J/cm^2^, could effectively reduce the inflammatory lesions of severe acne vulgaris patients within 12 weeks (51). Due to the good clinical efficacy and low recurrence rate, ALA-PDT has been reported as a better choice for the treatment of genital warts (52). It can effectively clear latent or subclinical human papillomavirus infections by reducing viral loads (52, 53). In conclusion, aPDT successfully eliminate various pathogenic microorganisms *in vitro*, and show excellent anti-infective efficacy in clinical infectious diseases.

## The effects of photosensitizer-based therapies on skin cells

**FIGURE 2:**
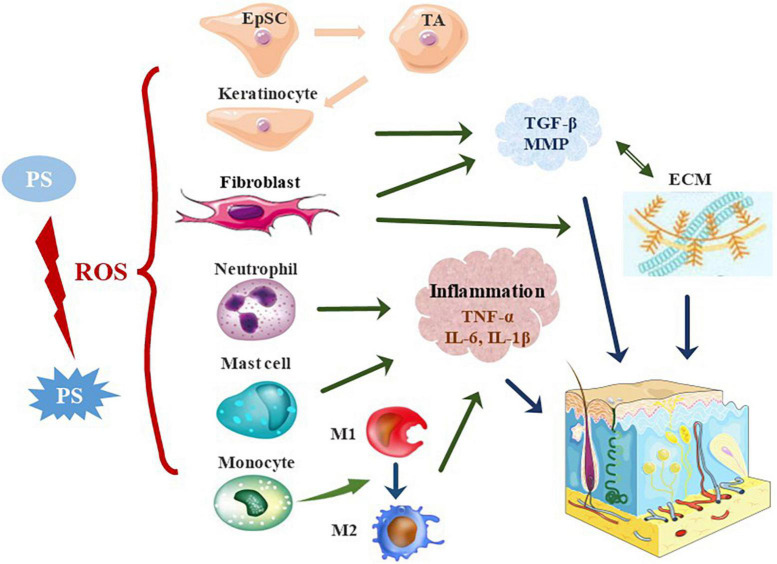
The effects of photosensitizer-based therapies on skin cells. In PDT, PS is activated to generate ROS, which promotes wound healing by stimulating skin resident cells and recruiting immune cells. PDT activates quiescent EpSCs to divide into proliferative TA cells, which promote re-epithelialization by enhancing their proliferation and migration. Besides, PDT stimulates the proliferation of keratinocytes and fibroblasts, and increases the secretion of TGF-β and MMP, thereby stimulating the production of collagen and elastin. PDT alters the local immune state and induces acute inflammatory responses in the early stages of trauma. PDT significantly affects the activation and migration of neutrophils. The neutrophil response is switched to that of monocyte-macrophages in the early stages of wound repair. The pro-inflammatory M1 phenotype is adopted in the early stages of injury and the anti-inflammatory M2 phenotype in the middle and late stages. PDT induces rapid recruitment of mast cells to further degranulate and release inflammatory molecules. EpSC, epidermal stem cells; TA cells, transient amplifying cells; ECM, extracellular matrix.

Wound healing after skin injury involves complex interactions among multiple cells, primarily keratinocytes, vascular endothelial cells, fibroblasts, recruited immune cells, and the related extracellular matrix (ECM), and all of them are indispensable in the process (Figure 2). The type and concentration of the PS and the light dose affect the effect of PDT on cells (54, 55). With the increase concentration of 5-ALA and energy density, the viability of normal fibroblast cell decreases (56). The light dose of 84 J/cm^2^ is quite safe for keratinocytes at concentrations ranging from 4 to 161 μM indocyanine green (ICG). Most keratinocytes were damaged at any concentration of ICG when light dose of 252 J/cm^2^ was used (55). By properly optimizing the energy density and PS concentration, the damage of PDT to related cells can be minimized (55, 56). Thus, researches in the field of oncology therapy involve higher concentration of PS to kill tumor cells, whereas PDT for antimicrobial and wound healing only requires lower dose.

### Resident cells

Under appropriate parameters, PDT could promote wound healing by directly affecting the functional behavior of skin resident cells (57–59). Significant proliferation of keratinocytes and fibroblasts, differentiation into myofibroblasts, and neovascularization after PDT have been observed in a number of studies *in vivo* and *in vitro* (60–65). PDT after injury appears to accelerate re-endothelialization and enhance the angiogenic response.

Re-epithelialization is a key event in wound healing, and it involves continuous proliferation, migration and differentiation of epidermal keratinocytes. The process requires the activation of quiescent epidermal stem cells (EpSC), which proliferate and migrate to the injured site and differentiate into stratified epidermis. Transient amplifying cells (TA cells) are the progeny cells of EpSC by asymmetric division with a proliferative capacity. ALA-PDT increased the proportion of TA cells and K14 expression. PDT could accelerate wound closure by enhancing EpSCs differentiation, proliferation and migration, thereby promoting re-epithelialization (66). 0.1 mM MAL irradiated with red light for 10 min (6.2 mW/cm^2^, 3.72 J/cm^2^) promoted the proper production of endogenous ROS, thereby effectively stimulating the proliferation of human immortalized keratinocytes, which may adjust the cell cycle process by activating Src kinase (67). PDT-conditioned medium containing interleukin 6 (IL-6) enhanced the migration of keratinocytes (68). ICG is a non-specific activator of the cystic fibrosis transmembrane conductance regulator. ICG-PDT conditioned medium collected from HaCaT cells exposed to 5 J/cm^2^ near-infrared light at 129 μM ICG activated cystic fibrosis transmembrane conductance regulator and enhanced the migration of HaCaT cells. The enhanced migration may be related to the activation of the cystic fibrosis transmembrane conductance regulator and the focal adhesion kinase pathway (69). Another study showed that ROS stimulated by ALA-PDT can inhibit the fibroblast growth factor receptor-2b pathway in downstream of protein kinase C, resulting in a decrease in IL-1α expression, and ultimately leading to keratinocyte differentiation and proliferation (70). Besides, PDT targeted to lysosomal membranes efficiently induced the differentiation of keratinocyte (71).

Fibroblast dysfunction is the key factor in the non-healing of chronic ulcers (72). Early activation of fibroblasts during PDT in ulcers has been observed (59, 62, 63). PDT at the ALA concentration of 0.1 mM, and light dose of 3 J/cm^2^ induced the production of intracellular ROS, thus it regulated prolonged extracellular signal-regulated kinase activation, and contributed to the fibroblast proliferation and activation (18). Fibroblast proliferation was enhanced by co-culture with keratinocytes treated with low-dose PDT (1 mM ALA, light dose of 1 J/cm^2^) (73). PDT with 38 μM ALA at light dose of 1 J/cm^2^ increased fibroblasts migration and closure of the scratch, which was more obvious in diabetic cells compared to normal cells (56). The proliferation and differentiation of fibroblast cells may be achieved by triggering phosphoinositol 3 kinase, protein kinase B phosphorylation, and extracellular signal-regulated kinase signaling pathways (60). Low-dose ALA-PDT (1 mM ALA, light dose of 1 J/cm^2^) can stimulate the transforming growth factor-beta1 (TGF-β1) in keratinocytes, which activated the TGF-β pathway in dermal fibroblasts to reshape the collagen in the dermis (73). Fibroblasts are an important source of the wound granulation tissue, and PDT enhances the functional activity of fibroblasts, thereby promoting wound repair.

The synthesis and degradation of the ECM are crucial in the process of wound remodeling. It is a highly dynamic structural network, which composes collagen, elastin, fibronectin, laminin and other glycoproteins. PDT apparently regulated the production of TGF-β during wound remodeling, which is significantly related to the reduction in the wound area (74–76). Skin wound healing model *in vitro* was used to evaluate the effects of PDT. The synthesis of collagen I and III was increased in the dermis around the healing wound, and the expression of matrix metallopeptidase 3 (MMP3), MMP19, p16, and α-smooth muscle actin (α-SMA) was significantly higher after treatment with 20% ALA and 20 J/cm^2^ of light dose (77). The increased expression of type I and III procollagen and MMP after ALA-PDT can be mediated not only by the direct effect on fibroblasts, but also by the indirect effect of keratinocyte-derived cytokines such as IL-1α and IL-6 (78, 79). In a photobiological stimulation study, the combined treatment with PS (aluminum phthalocyanine chloride) and light at intermediate dose (140 J/cm^2^) promoted collagen and elastin formation after 14 days, increasing up to 20% compared to the control sample. In addition, the expression of MMP-2 and MMP-9 increased accordingly (80). PDT induces changes in the ECM and its related molecules, which are essential for tissue repair.

### Immune cells

Upon wounding, skin resident cells recognize damage-associated molecular patterns and pathogen-associated molecular patterns, along with a series of signaling and secreted chemokines, leading to further inflammation and recruitment of other immune cells (81, 82). Recent studies indicated that PDT altered the local immune state (74), inducing acute inflammatory response in the early stage of wound healing and reducing chronic inflammation in the late stages (83). More specifically, PDT induced the recruitment of immune cells, such as neutrophils, mast cells, monocytes or macrophages, to the wound site, thereby facilitating the removal of damaged tissue from the wound and inducing pro-inflammatory cytokines.

Neutrophils are the first immune cells to reach the wound bed. PDT significantly affected the activation of neutrophils (84, 85). The infiltration of neutrophils reached a peak at 4 h. At this time, the expression of E-selectin was also increased significantly, and it was correlated strongly with the number of neutrophils (86). MB-PDT can increase the adhesion of neutrophils, but not modify the release of myeloperoxidase (87). Fibroblasts initially responded to PDT by expressing heat shock proteins, triggerring the nuclear factor kappa B pathway, prompting macrophages to secrete pro-inflammatory cytokines [tumor necrosis factor alpha (TNF-α), IL-6, and IL-1β]. The resulting immune response was believed to promote neutrophil migration (88). The activation of inflammatory cells and production of pro-inflammatory cytokines amplified the acute inflammatory response, facilitated wound cleansing and promoted subsequent tissue regeneration (89, 90). In addition to neutrophils, other innate immune cells such as monocytes, macrophages, and mast cells, are also activated and accumulated at the wound site immediately after PDT.

An experimental study observed that the neutrophilic response switched to the reaction of monocyte-macrophage cells in the early stage of wound repair (91). Monocytes translocated to the sites of damage and differentiate into macrophages upon activation. Macrophages play the key role in all stages of wound healing, adopt a pro-inflammatory M1 phenotype at the early stage of injury, while as anti-inflammatory M2 in the middle and later stages (90). A wide variety of cytokines are secreted to bridge the two roles (82, 90). It was found that the change in macrophages was consistent with the proportion of inflammatory factor (IL-1β, TNF-α)/growth factor (TGF-β1, vascular endothelial-derived growth factor) (83).

Mast cells are essential for controlling the bacterial load and wound healing (92). ALA-PDT could induce the rapid recruitment of mast cells, and cause further degranulation and release of inflammatory molecules, thereby stimulating the peripheral nerves to promote healing (93). A controlled study confirmed the important role of mast cells in PDT for ulcers, and immunohistochemical analysis revealed an increase in the number of mast cell, degranulation index, and the expression of the basic fibroblast growth factor (63). The increase of plasmacytoid dendritic cells, MHC-II expression, TNF-α positive mast cell expression, TGF-β expression, and CD4^+^/CD25^+^ regulatory T cells was observed on immunofluorescence staining in another study (74). Previous studies have shown that PDT cause beneficial activation of the innate immune system to promote wound healing, but its role in the adaptive immune response remains to be explored.

## The effects of the photosensitizer-based therapies on animal models

**FIGURE 3:**
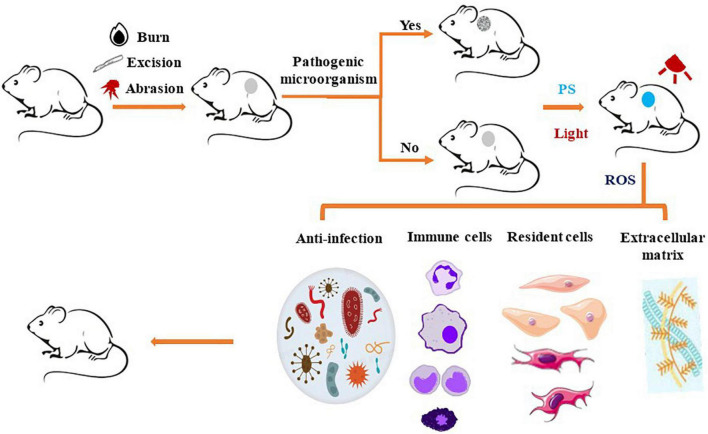
Schematic representation of photosensitizer-based therapies in animal wound model. The animals receive a burn, excision, or abrasion wound, which is uninfected or infected by pathogenic microorganisms. Next, PS is applied to the wound, then irradiated with a light source that transfers energy to generate ROS or heat, which remove various pathogenic microorganisms from wound surface. PDT induced the recruitment of immune cells, such as neutrophils, mast cells, monocytes or macrophages, to the wound site, thereby promoting removal of the damaged tissue from the wound and induction of proinflammatory cytokines. PDT also stimulates skin resident cells to promote re-epithelialization and deposition of extracellular matrix to promote wound healing.

Recently, the potential of PDT in tissue regeneration and wound healing has attracted the attention of many research groups. Before applying PDT to human clinical trials, many *in vivo* studies have been carried out using various animal models, including those of burns, excisions, and abrasions (Figure 3). It is worth noting that most of the wounds researched are infected. Bacterial infection will promote inflammation and produce virulence factors, which could aggravate the destruction of local tissue and delay healing (4, 6).

In a burn wound, recent study indicated that local and transient ROS production created by MAL-PDT (light dose of 2.5 J/cm^2^) could regulate the function of stem cells and tissues and activate cell proliferation in the niche of mouse skin and hair follicle stem cells, thereby promoting hair growth and wound healing (59). A study evaluated the effect of phototherapy on the healing process through clinical and histopathological analysis, and it showed the promotion of red laser, infrared, MB-PDT, and green LED on healing of third-degree burns in rats. Compared with untreated controls, PDT with 1.56 μM MB and 10 J/cm^2^ light dose stimulated the production and maturation of collagen, and the wound receded more (94). Another histological study in rats proved that PDT mediated by toluidine-O blue acted as a biostimulant adjuvant, balancing the adverse effects of burns during the wound healing process. It mainly acted on the early stage of healing, accelerated inflammation, promoted cell proliferation and chemotaxis, and increased collagen deposition. Besides, the formation of new blood vessels ranging from moderate to strong was also observed (95). The beneficial effect of phototherapy was obvious in the re-epithelialization and collagen matrix deposition, and the presence of PS accelerated this process. Smart hydrogel-based porphyrin photosensitizer sinoporphyrin sodium/basic fibroblast growth factor nanohybrids was developed and used for aPDT in the infected wound. Under mild light dose of 30 J/cm^2^, 8 μM material (quantified by sinoporphyrin sodium concentration) eradicated almost 99.99% of *S. aureus* and MRSA *in vitro* and promoted the healing of burn wound in mice (96). Whether the wound is infected or not, PDT is beneficial to the healing of burn wounds.

Excision is another common type of wound. Acute infection models in mice were established after full-thickness skin excision and then treated separately, it was found that the burden of *Pseudomonas aeruginosa* was significantly reduced in ALA-PDT group. In addition, further exploration of the mechanism showed that PDT not only killed bacteria, but promoted the healing of skin infection wounds by regulating inflammatory factors, affecting the polarization state of macrophages, and promoting granulation tissue formation, angiogenesis, collagen regeneration and remodeling (83). A study on wounds of diabetic mice showed that PDT with 10% ALA at light dose of 25 J/cm^2^ accelerated the rate of wound closure (97). After the excellent bactericidal properties of selenoviologen against *Escherichia coli* and *S. aureus* were verified *in vitro*, it was loaded into a hydrogel patch and exposed to 90 mW/cm^2^ white light for 60 min on infected excised wounds in mice. After 15 days, the wound area was significantly reduced in aPDT group. Researchers believed that the excellent antibacterial property created a crucial local and systemic immune microenvironment for tissue regeneration, thus playing an important role in skin regeneration process (98). Obviously, PDT exerts an anti-bactericidal effect on wound infection, which may promote wound healing.

A mouse model of abrasion inoculated with *C. albicans* was developed, and aPDT was performed with 400 μM phenothiazinium salt and red light at light irradiance of 32.5 mW/cm^2^. Whether aPDT initiated at either 30 min or 24 h after infection, the weakening of microbial luminescence and significantly reduced burden of *C. albicans* could be observed. This indicated that aPDT could be used to prevent and treat superficial *C. albicans*-infected wounds (99). Another study used MB-aPDT (light dose of 74 J/cm^2^ in 635 nm), MB-aPDT and daily topical mupirocin in combination or only mupirocin to treat superficial skin infection wounds in mice. Superficial contraction at 24 h was observed after MB-aPDT. Also, a larger area reduction at 48 h, faster crust detachment, fewer scales and no scars were noted. The results showed that MB-aPDT induced the best wound healing. Although the addition of mupirocin to MB-aPDT improved the antibacterial activity, it did not promote wound healing (100). Most studies on abrasion wounds focus on the antibacterial property of PDT and significant sterilization effect is observed after treatment.

Regardless of the type of wound, PDT can effectively promote healing. There was a synergetic effect among light, PS and delivery drug on tissue healing, and PDT will not cause any healing inhibition during the process (101, 102). However, there are some different opinions. Delayed wound re-epithelialization was observed in the second week of PDT treated with 20% MAL and 100 mW/cm^2^ of light irradiance. Immunohistochemical analysis revealed fewer CD31^+^ blood vessels after irradiation and no difference in collagen I density between treated and untreated wounds. Therefore, it was concluded that PDT delayed acute wounds healing in mice (103). This difference may be caused by the different power of the irradiation. Different studies used different PS and irradiation. Low-level irradiation was shown to have a stimulating effect, while high-level radiation could have an inhibitory effect on cells (54, 55, 104, 105). This study used a relatively large irradiance. In addition, the observation period was short, only 2 weeks. Other study indicated that PDT was associated with a smaller wound area in the remodeling period after 3 weeks (75). If the observation period could be extended, the results may be different.

## The effects of the photosensitizer-based therapies on human skin wounds

In view of the excellent sterilization effect shown by PDT *in vitro* and in animal studies, as well as the biostimulation of skin cells and promotion of wound healing, it has been applied to the study of human wounds. The clinical studies involve a variety of wounds, including chronic leg ulcers (venous, arterial, mixed, or diabetic), diabetic foot ulcers, acute excised wounds, and refractory ulcerative lesions of other diseases, such as cutaneous leishmaniasis (CL), lipid necrosis (NL), and pemphigus vulgis.

**FIGURE 4:**
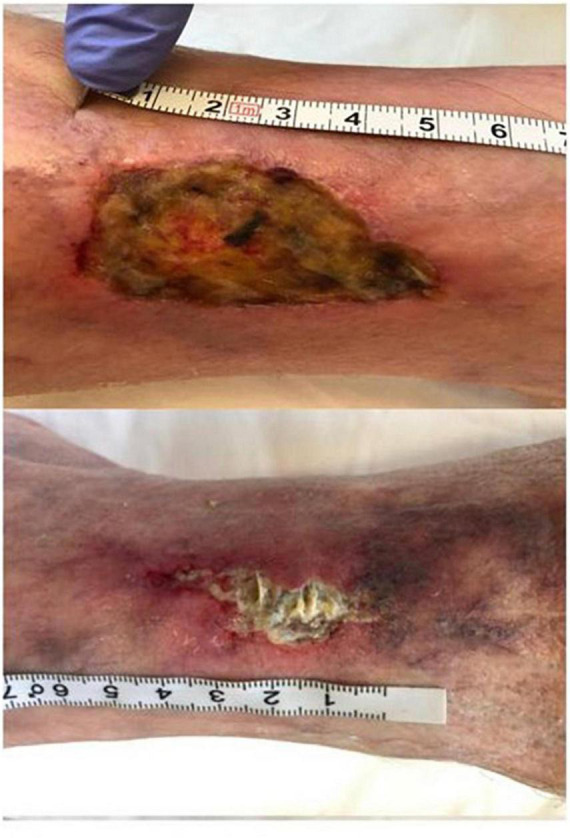
Aminolaevulinic acid-photodynamic therapy (ALA-PDT) for the infected leg ulcer. A patient with chronic foot ulcer before and after 3 cycles of PDT (figure adapted from the reference 107 with no change).

Chronic leg ulcers are common but challenging, and PDT is gradually applied to chronic leg ulcers in clinical practice (Table 1). More than one third of the studies in Table 1 (1st, 2nd, 3rd, and 6th) used 20% ALA as PS. Nineteen patients affected by venous leg ulcer (VLU) were managed with standard of care in addition to weekly ALA-PDT (light dose: 80 J/cm^2^). Wounds were significantly and gradually reduced after three sessions of treatment, and increased TGF-β was significantly associated with wound reduction (74). After PDT, mast cell degranulation index increased and the expression of neuron mediators increased. It was preliminarily suggested that ALA-PDT may promote the healing of VLU by stimulating quiescent peripheral nerves, possibly following the release of inflammatory molecules through granulated mast cells (93). Ten patients in the sixth study of Table 1 underwent 10 sessions of PDT (light dose: 80 J/cm^2^) with 20% ALA. At 8-month follow-up, four patients achieved complete remission, and four patients had more than 50% reduction in wound size (Figure 4) (106). To compare the efficacy of PDT using 20% ALA vs. red light alone (630 nm, 80 J/cm^2^) for chronic skin ulcers infected with *P. aeruginosa*, treated weekly with standard wet dressings during the treatment and routine care dressings for the rest time. PDT combined with care dressings demonstrated better antibacterial and healing effects (8). PDT containing 0.5% ALA and 0.005% EDTA-2NA (light dose: 10 J/cm^2^) was used to treat skin ulcers infected with MRSA and *P. aeruginosa*. Simple daily debridement was also allowed, and continuous PDT treatment for several days resulted in 40% reduction in wound area in six out of seven patients (10). 2% ALA was also applied to three cases of chronic ulcers that failed to respond to conventional treatment and the wounds healed after 1–3 sessions of PDT (20 mW/cm^2^ for 16.7 min, light dose: 20 J/cm^2^) without recurrence for more than 29 months (68).

**TABLE 1:** Clinical studies involving PDT in chronic leg ulcers.

Country	Design	Patients	Wounds	PS	Light	Treatment	Outcomes	Conclusion
Italy (74)	prospective cohort study	19 patients	VLU	20% ALA in liposomal gel	red light LED (630 nm, light dose: 80 J/cm^2^)	once a week up to 3 sessions	Wounds decreased progressively after three sessions of ALA-PDT, and the increase of TGF-β was associated with the reduction of wounds.	ALA-PDT combined with standard care has beneficial effects on chronic VLU.
Italy (93)	prospective cohort study	19 patients	VLU	20% ALA in liposomal gel	red light LED (630 nm, light dose: 80 J/cm^2^)	1 session	The mast cell degranulation index and cellular the expression of various neuronal mediators increased after ALA-PDT.	ALA-PDT may promote VLU healing by stimulating quiescent peripheral nerves, possibly after degranulation of mast cells.
China (8)	randomized controlled trial (RCT)	26 patients	chronic leg ulcers infected with *P. aeruginosa*	20% ALA solution, incubate for 1.5 h in the dark	red light (light dose: 80 J/cm^2)^	once a week for 2 sessions	Wound bacterial load was significantly reduced at 24 h after PDT. Wound area reduction in PDT group was more significant than the control group at 7 days after treatment.	PDT is a potential modality to control infection and promote chronic ulcers healing.
Japan (10)	case series	7 patients	leg ulcers infected with MRSA and *P. aeruginosa*.	macrogol ointment containing 0.5% ALA-HCl and 0.005% EDTA-2Na	LED light (410 nm, light dose: 10 J/cm^2^)	repeat once daily	Wound size was reduced more than 40% in 6 of 7 patients, and ALA-PDT was considered safe in all patients.	ALA-PDT is safe and effective for MRSA and *P. aeruginosa* infected skin ulcers.
China (68)	case series	3 patients	chronic leg ulcers	2% 5-ALA solution	PDT-1200 lamp (20 mW/cm^2^ for 16.7 min, light dose: 20 J/cm^2^)	1–3 sessions	The ulcers healed after 1 to 3 sessions PDT, without recur for more than 29 months, and no bacteria were isolated after treatment.	ALA-PDT may be an effective treatment for patients with refractory infected ulcers.
Poland (106)	RCT	20 patients (10 in the treatment group 10 control)	chronic and infected leg ulcers	20% ALA/Octenilin gel, incubate for 4 h	laser light (630 nm, light dose: 80 J/cm^2^)/placebo: mimickedred light	3-week interval for 3–5 sessions	After 8 months of follow-up, 40% patients in PDT group achieved complete remission, 40% obtained partial remission (wound size reduction >50%), and 10% had no response.	PDT can be used to treat chronic ulcers, as a minimally invasive and effective method.
United Kingdom (119)	case report	a 72-year-old woman	VLU	5-ALA	red light (633 nm)	twice a week for 8 sessions	Significant improvement in ulcer was observed after PDT, with negative skin swabs.	ALA-PDT seem to be an alternative therapeutic strategy for VLU.
Italy (108)	case series	9 patients	VLU	4% MAL, incubate for 45 min	red light (630 nm, light dose: 18 J/cm^2^)	3-week intervals for a mean of 8 sessions	The ulcers healed in all patients in an average of 24 weeks and no ulcer recurrence after PDT.	MAL-PDT can be effective in the treatment of VLU.
Brazil (109)	RCT	12 patients, control (*n* = 6) and PDT (*n* = 6)	diabetic ulcers in lower limbs	0.01% MB	red laser (660 nm, 30 mW/0.04 cm^2^, 8 s, 6 J/cm^2^ per point)	three times a week for 10 sessions	Two methods of measurement showed a greater reduction in wound size in PDT group compared to the control group.	PDT accelerates wound closure and can be tracked by different measurement methods.
Italy (107)	observational study	36 patients (13 males, 23 females)	infected leg ulcers of different pathophysiology	RLP068, incubate for 0.5 h	a portable LED light (630 nm, 8 min, light dose: 60 J/cm^2^)	2 sessions	Single PDT was effective in reducing bacterial load, and almost all bacterial swabs of ulcer were negative after the second treatment.	RLP068-PDT is effective in reducing bacterial load in patients with infected leg ulcers.
Brazil (120)	case report	50-year-old men	chronic leg wounds	1.5% curcumin emulsion, incubate for 0.5 h	LED (450 nm, 75 mW/cm^2^ for 12 min, light dose: 54 J/cm^2^)	twice a week for 8 sessions. 2 days latter, LLLT at 660 nm.	The wound area decreased progressively during treatment.	The combined treatment is effective for the healing of arterial and venous ulcers, and can be considered as a promising treatment.

**FIGURE 5:**
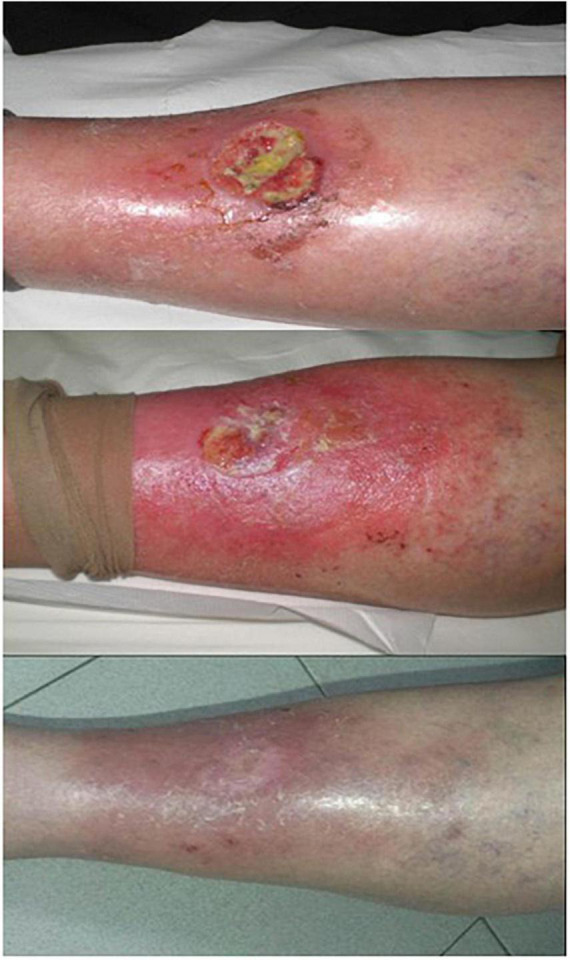
Effective use of PDT on chronic leg ulcer. Up: a 67 years old woman presenting a chronic leg ulcer on the left tibia. Middle: almost complete healing of the ulcer after five treatments (15 weeks). Down: complete healing of the ulcer in 12-month follow-up (figure adapted from the reference 109 with permission of John Wiley and Sons).

Other types of PS have also been applied to the treatment of chronic leg ulcers. RLP068-PDT (total light dose: 60 J/cm^2^) was used to treat 36 cases of infected leg ulcer with different pathophysiology. Except for four patients who reported severe pain, other patients were well tolerated. The results showed that PDT could effectively reduce bacterial load without any systemic or local antibiotics, ensuring the smooth progress of follow-up treatment (107). VLU was treated with 4% MAL combined with red light (630 nm, light dose: 18 J/cm^2^), and PDT was performed at 3-week interval. After treatment, the ulcers were covered with cold water and chamomile-soaked gauze and aloe vera gel twice a day for 10 days. All the patients healed from ulcers completely in a mean of 24 weeks (Figure 5) (108). Macromorphometric assessments using two measurements to track the repair process showed that MB-PDT (0.01% MB, 6 J/cm^2^) in combination with conventional drug (collagenase/chloramphenicol) accelerated wound healing (109). Combination therapy with PDT based on different PS has beneficial effects on chronic leg ulcers.

Diabetes foot ulcer (DFU) is an increasingly serious public health problem, and the treatment is very difficult. Infection is the most common complication associated with delayed healing and increased risk of amputation (110). The prognosis of DFU treated with PDT combined with systemic antibiotics was better (111). The clinical study of PDT on DFU is shown in Table 2. A randomized, double-blind, placebo-controlled phase IIa trial was conducted in 62 patients with DFU. PDT (light dose: 60 J/cm^2^) with different concentrations of RLP068 (0.10, 0.30, and 0.50%) was used as a supplement to the systemic treatment of amoxicillin and clavulanic acid. The results showed that RLP068-PDT as add-on to systemic antibiotic treatment reduced the bacterial load, and the total microbial load decreased in a dose-dependent manner (112). In a multicenter study, 64 patients with DFU received repeated PDT (red light irradiation for 8 min, 60 J/cm^2^) to study the effect of RLP068 on bacterial load and ulcer healing. After a single treatment, the bacterial load was significantly reduced, and the benefit lasted for 2 weeks. During follow-up, even patients infected with gram-negative bacteria or exposed bones had a significant reduction in ulcer area (113). 16 patients with chronic leg ulcer and 16 patients with DFU were recruited into a placebo-controlled, single treatment phase IIa trial. 0.026% PPA904 or placebo was applied topically and immediately irradiated with 50 J/cm^2^ red light. Both groups received standard bandaging therapy. All patients were well tolerated. Compared with placebo, patients showed a reduction in bacterial load immediately after PDT. After 3 months, 50% patients of PDT group healed completely, while only 12% in placebo group. A significant reduction in bacterial load and a clear trend in wound healing was observed in the study (50). However, Phase IIa clinical studies are mainly exploratory trials of efficacy with a small number of subjects included. No Phase III clinical study with more cases has been reported yet. Further researches in this area need a larger number of patients.

**TABLE 2:** Clinical studies involving PDT in DFU.

Country	Design	Patients	PS	Light	Treatment	Outcomes	Conclusion
Italy (112)	RCT	62 patients with type 1 or type 2 diabetes	0.10, 0.30, or 0.50% RLP068 gel or placebo	red light (689 ± 5 nm, 500 s, light dose: 60 J/cm^2^)	one session	A dose-dependent reduction in microbial load was observed immediately after PDT, with a progressive fading during follow-up.	RLP068-PDT produces a significant reduction in bacterial load, as a supplement to systemic antibiotic therapy.
Italy (121)	case series	4 patients affected by DFUs	RLP068, incubate for 0.5 h	LED light device (630 nm, 8 min, light dose: 60 J/cm^2^)	twice a week	RLP068-PDT reduced the microbial load in DFU, and wound size reduced significantly after several sessions treatment.	RLP068-PDT could be used as an add-on to antibiotic therapy to eradicate infection and promote healing.
Italy (122)	case series	22 patients (15 males, 7 females)	RLP068, incubate for 0.5 h	red light LED (630 nm, 8 min, light dose: 60 J/cm^2^)	twice a week, ranged from 4 to 16 sessions	68% of the patients healed or the wound size was reduced by >50%.	RLP068-PDT could improve some chronic ulcers that are resistant to other anti-infective methods.
Italy (113)	multicenter, retrospective study	64 patients with diabetes	RLP068, incubate for 0.5 h	red light (630 nm, 8 min, light dose: 60 J/cm^2^)	twice or thrice a week	Bacterial load reduced significantly after single PDT, and the effect lasted for 2 weeks, and the wound size significant reduced.	RLP068-PDT seems to be a promising topical wound management for infected DFU.
Italy (9)	case series	17 persons with diabetes	RLP068	red light (630 nm, light dose: 60 J/cm^2^)	twice a week for 8 sessions	In all cases, PDT was found to reduce wound size and decrease the bacterial load.	RLP068-PDT is effective for faster healing of DFU.
Brazil (111)	controlled clinical trial	34 (16 in the control and 18 in the treatment group)	1% MB and O-toluidine blue in aqueous solution	white halogen light (10 mW/cm^2^, 10 min, 6 J/cm^2^)	twice a week	The rate of amputation in PDT group was 0.029 times of the rate in the control group and the difference is statistically significant.	PDT combined with systemic antibiotics provides a better prognosis for diabetic feet.
United Kingdom (50)	RCT	32 patients (16 patients with chronic leg ulcers;16 with DFU)	0.026% PPA904 or placebo, incubate for 15 min in the dark	red light (570–670 nm, light dose: 50 J/cm^2^)	1 session	The bacterial load decreased immediately after PDT. After 3 months, 50% patients of PDT group healed completely, while only 12% in placebo group.	The trend of healing and the bacterial load reduction was observed in chronic wounds after PPA904-PDT.

Photodynamic therapy has also been applied to other types of ulcers (Table 3). MAL-PDT (irradiance by 80 mW/cm^2^ red light for 9 min, total light dose: 37 J/cm^2^) affected the healing of human skin excision wounds. Resecting the wound during the inflammatory phase, matrix remodeling and cosmetic effects/skin structure showed that wound re-epithelialization delayed at 7 days after MAL-PDT, with smaller wounds at 3 weeks, and greater and more ordered collagen and elastin deposition at 9 months (75). These results suggested that MAL-PDT had a positive stimulation of the inflammatory phase, matrix remodeling, and ultimately producing improved dermal matrix architecture.

**TABLE 3:** Clinical studies involving PDT in other types of ulcerations.

Country	Design	Patients	Wounds	PS	Light	Treatment	Outcomes	Conclusion
United Kingdom (75)	RCT	27 healthy older men	4-mm punch biopsy wound in upper inner arm	16% MAL, incubate for 3 h	red light (80 mW/cm^2^ for 9 min, total light dose: 37 J/cm^2^)	wounds was treated immediately and again on days 2 and 4	Wound re-epithelialization delayed at 7 days after MAL-PDT, with smaller wounds at 3 weeks, and greater and more ordered collagen and elastin deposition at 9 months.	MAL-PDT acts on the matrix remodeling process and ultimately improves wound appearance
Italy (123)	case report	a 38-year-old woman with SLE and APS	two symmetric paramalleolar ulcers	10% ALA in PEG ointment, incubate for 2 h	red light (630 nm, 160 mW/cm^2^, 8 min, light dose: 75 J/cm^2^)	the left ulcer dressing only, the right with dressing and PDT (once a week)	The ulcers healed within 3 months leaving depressed white scars, and have not recurred since.	PDT may be a valid alternative strategy to control the ulcers caused by systemic vascular and immunological process.
Greece (124)	case report	a 69-year-old patient with CL	an ulcerated lesion on the left cheek	20% 5-ALA solution, incubate for 4 h in the dark	red light (630 nm, 45 mW/cm^2^, light dose: 100 J/cm^2^)	once a week for 3 sessions	The lesion had subsided and remain clinically clear at 2 years follow up.	ALA-PDT may be an alternative treatment in recurrent CL.
Israel (114)	single-center controlled study	31 patients with CL	leishmanial lesions	16% MAL, incubate for 0.5 h	daylight for 2.5 h	once a week until cure	The overall cure rate for DA-PDT was 89, 86% in hospital group and 92% in the self-administered group.	DA-PDT is effective in the treatment of CL.
Denmark (115)	case report	a 15-year old boy with CL	a single, ulcer on the leg	BAF 200 ALA	−	twice a week for 12 weeks	Repeated ALA-PDT resulted in complete healing of the ulcer.	The complete healing of the ulcer suggests the utility of PDT in resistant cases of CL.
Italy (116)	case report	a 44 year old woman with NL	widespread ulcerative NL	10% ALA in polyethylene glycol ointment, incubate for 3 h	red light (630 nm, 160 mW/cm^2^, 8 min, light dose: 75 J/cm^2^)	2 weeks intervals for 6 sessions	The ulcers were completely healed, and the erythema was significantly reduced after 6 sessions of treatment.	PDT-stimulation of wound healing may influenced the course of NL positively.
Brazil (117)	case report	A 30-year-old woman with pemphigus vulgaris	persistent neck ulceration	MAL, incubate for 3 h	LED (635 nm, 10 min, light dose: 37 J/cm^2^)	once a week for 2 sessions	Two sessions of MAL-PDT healed the lesion completely.	MAL-PDT may be a useful adjunct in the management of recalcitrant ulcers in patients with pemphigus vulgaris.
China (118)	case series	Seven patients	skin ulcers with sinus tract formation	20% ALA in an oil-in-water emulsion	a red LED (633 nm, 84 mW/cm^2^, light dose: 100 J/cm^2^)	every 10 days for 1–5 sessions.	Six patients completely cured after PDT combined with antibiotics for 3 months. In another case, the sinus tract healed and wound size reduced.	ALA-PDT combined with antibiotics is safe and effective to treat skin ulcers with sinus tract.

**FIGURE 6:**
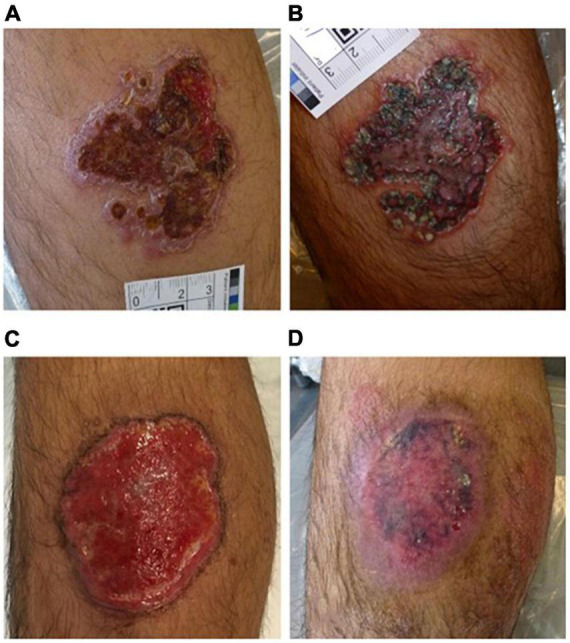
Effective treatment with PDT of cutaneous leishmaniasis. **(A)** Original lesion before treatment, **(B)** after oral fluconazole and topical ketoconazole, **(C)** after 12 PDT treatments, **(D)** 1 month after PDT treatment (figure adapted from the reference 116 with permission of John Wiley and Sons).

**FIGURE 7:**
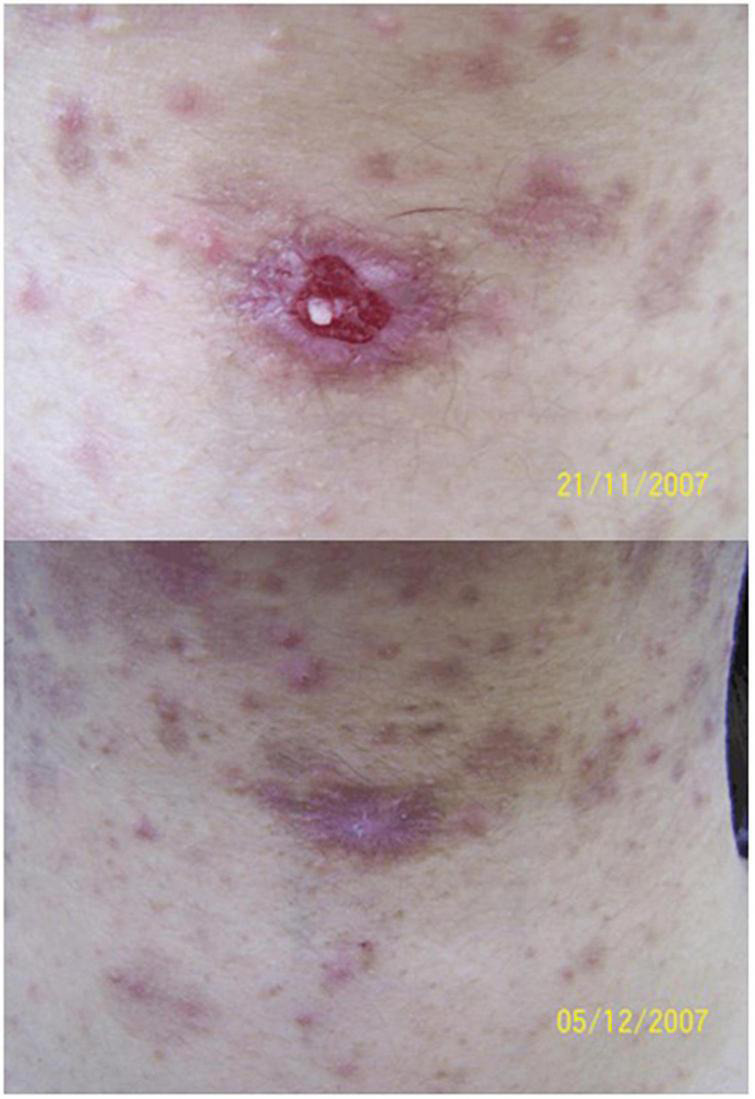
Methyl aminolaevulinate-photodynamic therapy (MAL-PDT) for recalcitrant ulcer in pemphigus vulgaris. Up: recalcitrant ulceration on the cervical region in a patient with pemphigus vulgaris. Down: complete closure of the ulceration 1 week after two sessions of MAL-PDT (figure adapted from the reference 118 with permission of John Wiley and Sons).

The main manifestation of CL was scar skin ulcer. 31 patients with CL received daylight activated PDT (DA-PDT). Fourteen patients were treated in the hospital, coated with 16% MAL, and then exposed to the sun for 2.5 h. Others received self-management treatment at home. The treatment was repeated weekly until clinical and microbiological cure. The total cure rate was 89%, DA-PDT had been proved to be effective in the treatment of CL (114). A 15-year-old boy with leg ulcer received PDT. He was previously resistant to pentavalent antimony, oral fluconazole and local ketoconazole. His ulcer healed completely after repeated ALA-PDT (Figure 6) (115). Compared with systemic therapy, this method should be considered as an attractive antiparasitic treatment, especially for drug-resistant cases. A 44-year-old woman with extensive ulcerative NL received local ALA-PDT (10% ALA, 160 mW/cm^2^ of light irradiance for 8 min with total light dose of 75 J/cm^2^) every 2 weeks. At the end of six sessions, complete healing of skin ulcer was observed, and erythema was significantly improved in all treatment sites (116). MAL was applied to the persistent ulcer in the neck of patients with pemphigus vulgaris. After two sessions of PDT (635 nm, light dose: 37 J/cm^2^), it healed completely (Figure 7) (117). MAL-PDT may be a useful adjunct for the treatment of refractory ulcer in patients with pemphigus vulgaris.

The formation of sinuses in chronic skin ulcers complicates the treatment. Seven patients with skin ulcers with sinus tract formation received PDT with 20% ALA every 10 days. They had previously received systemic antibiotic therapy, debridement and dressing change treatment, and there was no significant improvement. The light irradiance was 84 mW/cm^2^, and the light dose was 100 J/cm^2^. After 1–5 sessions, six patients completely cured after receiving the combination of ALA-PDT and antibiotics. In another patient, the sinus cured and the ulcer area reduced. There was no serious discomfort during the treatment, and all patients were satisfied with the effect (118). ALA-PDT combined with antibiotics decreases the treatment time for skin ulcers and sinuses, reduces the pain of patients, and it has become a promising method for the treatment of refractory skin ulcers and sinuses.

It is worth noting that all above studies show the benefit of PDT on wound healing, especially for some complex and difficult-to-heal chronic ulcers, which are usually resistant to conventional treatments. However, some limitations should be recognized. The clinical data are not solid. Some studies are only case report or case series. The sample size is small and RCT studies are limited. Different PS are used, such as ALA, MAL, MB, curcumin, etc., the treatment schemes vary, treatment cycles ranging from one to several times. Some studies use PDT in combination with standard care, some with antibiotics, and some apply PDT alone. No standardized treatment plan yet. Lack of comparative study on the efficacy of PDT with different PS. In the future, more large samples and multicenter clinical studies are needed to explore the role of PDT in wound healing.

## Conclusion

Besides the excellent sterilization effect, PDT can amplify acute inflammation, stimulate skin resident cells to proliferate, migrate and secrete cytokines, accelerate the proliferation of blood vessels and the deposition of ECM (elastin and collagen), thereby promoting the skin regeneration process. However, various PS have been used in different *in vitro* experiments and animal experiments, only several PS have been used clinically. In the future, more research is needed to explore the more detailed molecular mechanism of this therapy, develop a wider variety of PS, and explore clinical drug regimens to ensure the maximum efficacy and safety of this effective clinical tool. In view of its excellent antibacterial properties and its role in promoting wound healing, the emergence of PDT will reduce the application of antibiotics, slow down the emergence of drug-resistant strains, and provide a promising weapon for the treatment of refractory skin ulcers.

## Author contributions

XN wrote the manuscript and created the figures. WZ and GH revised the manuscript. YX conceived and revised the manuscript. All authors reviewed the manuscript.

## Abbreviations

PDT: photodynamic therapy
aPDT: antimicrobial photodynamic therapy
PTT: photothermal therapy
ROS: reactive oxygen species
PS: photosensitizer
MRSA: methicillin-resistant *Staphylococcus aureus*
MB: methylene blue
LED: light-emitting diode
ALA: aminolaevulinic acid
MAL: methyl aminolaevulinate
ECM: extracellular matrix
ICG: indocyanine green
EpSC: epidermal stem cells
TA cells: transient amplifying cells
MMP: matrix metallopeptidase
IL: interleukin
TGF- β: transforming growth factor- β
α-SMA: α-smooth muscle actin
TNF- α: tumor necrosis factor alpha
CL: cutaneous leishmaniasis
NL: necrobiosis lipoidica
VLU: venous leg ulcer
DFU: diabetic foot ulcer
DA-PDT: daylight-activated photodynamic therapy
RCT: randomized controlled trial.
